# The “Pelvic Harness”: a skeletonized mesh implant for safe pelvic floor reconstruction

**DOI:** 10.1590/S1677-5538.IBJU.2015.0177

**Published:** 2016

**Authors:** Sumerova Natalia, Neuman Menahem, Krissi Haim, Pushkar Dmitri

**Affiliations:** 1 European Medical Center, Moscow, Russia;; 2 Urogynecology Unit, Departaments of Obstetrics and Gynecology, Galilee Hospital, Nahariya;; 3 Bar-Ilan University, Faculty of Medicine, Safed, Israel;; 4 Helen Schneider Hospital for Women, Rabin Medical Center, Petach Tikva, and Sackler Faculty of Medicine, Tel Aviv University, Tel Aviv, Israel;; 5 Department of Urology, Moscow Medical Stomatological University, Russia

**Keywords:** Reconstructive Surgical Procedures, General Surgery, Pelvic Floor Disorders

## Abstract

**Objectives:**

To evaluate the feasibility, safety and surgical results of skeletonized mesh implants to form a pelvic harness for pelvic floor reconstruction surgery.

**Study design:**

Patients with advanced pelvic floor prolapse were enrolled to this study. Study model was a kit mesh, reduced to 75% of the original surface area by cutting out mesh material from the central mesh body. Patients were evaluated at the end of the 1^st^ and 6^th^ post-operative months and interviewed at the study conclusion.

**Results:**

Ninety-five women with advanced pelvic floor prolapse had this implant. Mean follow-up duration was 9 months (6-12 months). The POP-Q point’s measurements showed marked and statistically significant improvements. Bladder over-activity symptoms, fecal incontinence, pelvic pain and constipation rates were all reduced as well. No adverse effects related to the dissection or mesh implantation were marked. The first and sixth post-operative month follow-up records as well as the study conclusion interview findings were satisfactory in terms of subjective and objective cure and adverse effects occurrence.

**Conclusion:**

This study data proposes that skeletonizing meshes might be safely and successfully implanted for potentially improved pelvic floor reconstruction.

## INTRODUCTION

Pelvic organ prolapse (POP) is a common condition negatively affecting the quality of life of millions of women worldwide, with a lifetime prevalence of 11% (1). Women with advanced symptomatic POP experience daily discomfort, as well body image dissatisfaction and impaired sexual function (2). Treatment for POP requires significant health care resources (3), with an ever-growing impact in parallel with the growing elderly population (4,5).

According to recent studies, approximately one in ten women will undergo surgery for POP and/or incontinence during their lifetime (6). Many favor the trans-vaginal route over the abdominal approach; hence, the vagina is widely accepted as the natural orifice for POP reconstruction. Yet, POP repair surgeries have an unacceptably high failure rate with a 10-year reoperation rate of 17% reported by some (7) and disagreed by others (8). This may be attributed to weakness of fascial tissue, related to genetic factors, reduced collagen content or increased collagen destruction (9).

In an attempt to reduce these high failure rates, synthetic meshes were designed and implanted. They provided reinforcement and better support for vaginal surgical repair of prolapse. This led to a significant reduction in anatomical failure and reoperation rates (10, 11). However, mesh implant-related complications ranged from mild issues of transient pain and small mesh erosions to severe adverse effects such as large vaginal mesh exposures or extrusions, perforations into the bladder or bowel, and chronic pain. Mild mesh complications can be managed conservatively, but bladder or bowel injuries, fistulae, abscess formation, and debilitating pain may require repeat surgery and are not always curable (12).

One of the recent implant modifications aimed at reducing adverse effects is the partial absorbable mesh (13-15). It is assumed that significant reduction of the implant mass may lead to reduction of the adverse effects and complications of the graft that are thought to be directly related to the mesh mass.

The current pelvic floor implant meshes are designed to cover the whole pelvic floor area, even though the native pelvic floor architecture is more ligamentary rather than a sheath-like. This leads to creation of large surface area of the mesh implants and to potentially increased rate of mesh related post-operative complications. The most significant mesh complications are erosions and post-operative pelvic pain which probably are directly related to the implant total mass and surface area. As mesh surgery is an important surgical tool for pelvic floor reconstruction, it is important to look for new ways to reduce the mesh complication rates.

**Figure 1:**
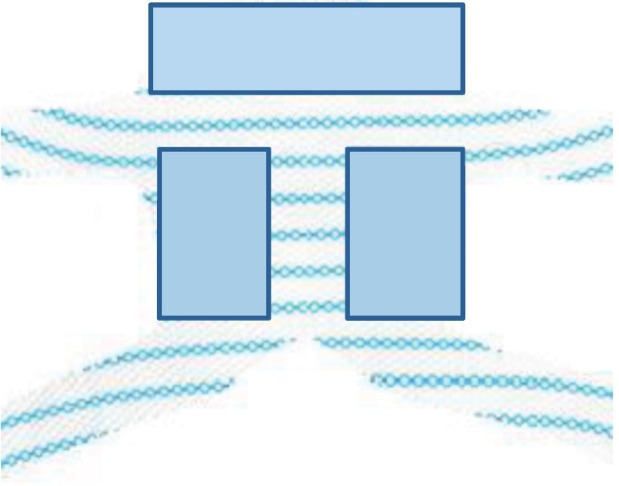
Anterior mesh surface area reduction.

In this study we suggest skeletonization of the common used pelvic floor mesh implants and making that structure more ligamentary than fascial by a significant reduction of the total implant mass, to create a pelvic harness rather than sheath-like implant (Figure-1).

The aim of this study was to evaluate the feasibility, safety and short-term surgical results of skeletonized mesh implants for pelvic floor reconstruction surgery.

## MATERIALS AND METHODS

We conducted a prospective observational study following women, scheduled to undergo reconstructive pelvic surgery for symptomatic and advanced anterior or posterior pelvic floor compartment prolapse, using trans-vaginal mesh implants, during January 2012 to March 2013. The local IRB approved the study protocol.

All women experiencing symptomatic stage 3 vaginal wall prolapse, and being at increased risk for prolapse recurrence, who have been scheduled for POP vaginal reconstruction with a mesh implant were included in the study, after having a meticulous explanation regarding the potential benefits and adverse effects of having a small mesh implant. Risk factors for prolapse recurrence included previous POP reconstructive surgery and clinical assessment of poor pelvic floor tissue. Exclusion criteria were pelvic inflammatory disease and chronic pelvic pain.

Prior to surgery, all patients completed a comprehensive questionnaire on symptoms of prolapse, urinary, bowel, and sexual malfunction. Preoperative evaluation included a detailed pelvic site-specific vaginal examination at lithotomy position with a Sim’s speculum during a maximal Valsalva maneuver and Pelvic Organ Prolapse Quantification (POP-Q) measurements and staging according to the standardized International Continence Society (ICS) scoring system (16). Each compartment (apical, anterior and posterior) was separately evaluated for detection of defects in pelvic support.

Patients underwent trans-vaginal mesh placement using the partially absorbable mesh Gynecare Prolift+M (Ethicon, Summerville, USA), minimized by mesh body cutting down of 75% of the mesh surface, giving it a skeletonized ligamentary harness appearance. Anti-incontinence surgery was performed when indicated using sub-mid-ureteral synthetic tape, according to the surgeon’s preference.

All patients were administered first generation Cephalosporin 1g intravenously, half an hour before surgery. An iodine antiseptic wash was applied to the area prior to the onset of surgery. All procedures were performed under general anesthesia. The detailed surgical technique was as published before (17). This included 50 milliliter saline hydro-dissection at the mid-line of the affected compartment vaginal wall, longitudinal incision, sub fascial lateral dissection towards the pelvic side wall up to the iliac spine and then to the mid-portion of the sacro-spinous ligament, through pass of the needle guide and the mesh arm thereafter. The other pair of arms was passed through the obturator plate for the anterior compartment or through the para-anal area for the posterior compartment reconstruction. The reduced mesh was placed and flattened, and the vaginal wall was re-sutured by two layers: first fascia and then mucosa with running absorbable sutures.

At the end of the 1^st^ and 6^th^ postoperative months, all patients were asked to complete the same questionnaire they had been given before surgery, and patients were re-evaluated with site-specific vaginal pelvic examination. Postoperative pain was assessed with the visual analogue scale (0-10) where 10 indicate maximal pain.

At the study conclusion, patients were interviewed by telephone for possible mesh-related complications and pelvic floor symptoms. The primary outcome measure was the mesh implant adverse effects, and the secondary outcome measure was the subjective cure rate, among the patient group.

Statistical analysis was performed with Vassar Stats Statistical Computation. The Wilcoxon signed-ranks test was used to evaluate quantitative parameters data distribution among groups.

Point bi-serial correlation coefficient was used to calculate P values for changes from baseline to postoperative parameters. Significance has been set for a value of P<05.

## RESULTS

**Figure 2:**
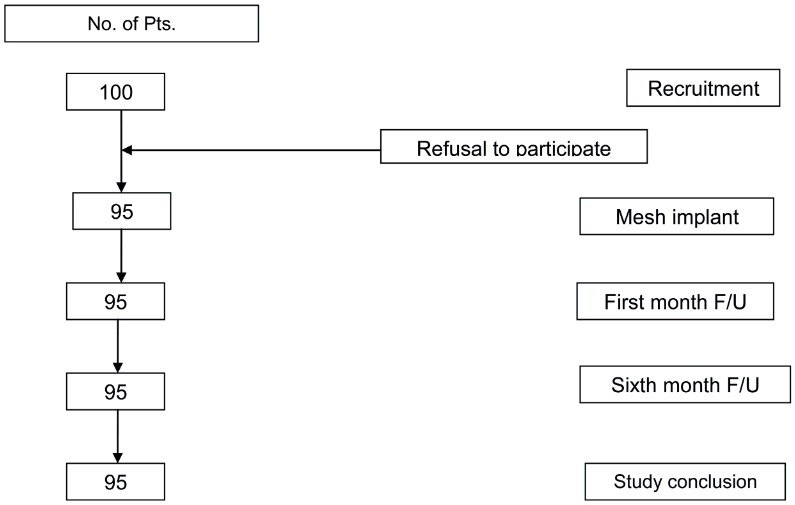
Patient flow-chart

Of the 100 women enrolled in this study, 5 refused participation after having a thorough informed consent presentation, while 95 (95.0%) accepted participation and underwent surgery using the skeletonized non-absorbable mesh implants from January 2012 through March 2013 (Figure-2). The mean age was 64.5±9.0 year (range 43-83); all patients had advanced anterior or posterior wall prolapse, and all were admitted for corrective surgery with the skeletonized mesh implants. The mean follow-up duration was 9.5±3.8 months (6-12 months). Patient’s characteristics are shown in Table-1. Only 17 women (17.9%) had previous POP surgery and 16 (16.84%) had previous hysterectomy. All patients had anterior mesh, 46 women (48.4%) had also posterior pelvic floor reconstruction (21 with mesh implants) and 33 (34.7%) had a concomitant anti-incontinence procedure.

**Table 1:** Patients characteristics (N = 95).

Variable	Mean±SD
Age	64.5±9.0 (Rang:43-83 Year)
Parity (no.)	2.6±1.6
BMI	24.8±2.4
Previous hysterectomy	16 (16.8%)
Previous POP surgery	17 (17. 9%)
Background chronic illness	35 (36.8%)
Follow-up duration	9.5±3.8 (6-12 Mnt)
Concomitant posterior compartment mesh*	25 (26.3%)
Concomitant posterior compartment colporrhaphy	21 (22.1%)
Concomitant anti USI operation**	33 (34.7%)

**POP** = Pelvic organ prolapse; **USI** = Urinary stress incontinence; **Mnt** = Month

* 12 skeletonized Prolift +M, 12-Prosima, 1-Elevate

** 22-TVTA, 9-TVTS, TVTO-2

Mesh implementation and placement was feasible in all cases. Regarding the primary outcome measure, there was not a negative effect of the skeletonization or any difference in insertion than the original mesh procedure. The perioperative complications are summarized in Table-2. No major complications were noticed, viscera were not injured, blood transfusion was not indicated, pain and infection rate and severity were modest. No adverse effects related to the dissection or mesh implantation were marked.

**Table 2:** Operative details.

	No. (%)	Outcome
Successful mesh placement	95 (100%)	
Urinary, bowel or ureteral injury	0 (0.0%)	
Operative bleeding > 200 ml	4 (4.2%)	No blood transfusion
Hematoma	1 (1.0%)	Self-resumed
Late post-operative pelvic pain	1 (1.0%)	Surgical mesh arm release at OR
Gluteal abscess	1 (1.0%)	Antibiotics
Granulation tissue	1 (1.0%)	Surgical removal at the outpatient clinic
Cervical elongation	1 (1.0%)	Partial cervical amputation

**Figure 3:**
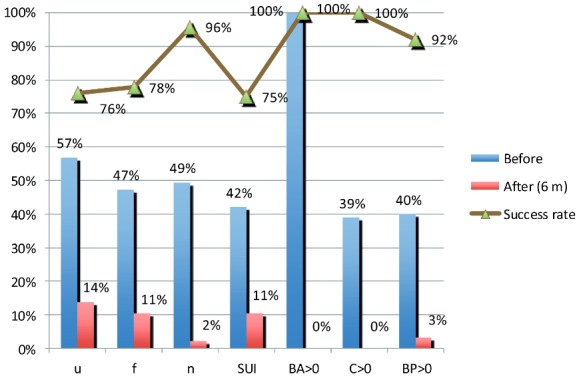
Objective and subjective success rate

The postoperative POP-Q measurements showed marked statistically significant improvements: the average delta for the POP-Q Ba point was 7.51cm, for the Bp point it was 2.69cm, and for the C point the delta was 6.72cm. The secondary outcome measures, including the subjective and objective cure rates, urinary, sexual and defecation functions are shown in Table-3. Bladder over-activity symptoms, namely urgency, frequency and nocturia, were all found to be reduced significantly. Faecal incontinence, pelvic pain and constipation rates were reduced as well. The first and sixth post-operative month’s follow-up records as well as the study conclusion interview findings were satisfactory in terms of subjective and objective cure and adverse effects occurrence. There was a statistically significant improvement in bladder over-activity symptoms and stress urinary incontinence. There were only two cases (2.1%) of dyspareunia not significantly different from preoperative rate. The overall subjective and objective outcome results of this study are promising (Figure-3).

**Table 3:** Patients outcome.

Variable	Prior to surgery	First Post-Op Mnt	Sixth Post-Op Mnt	P value
Urgency	54 (56.8)	10 (10.5)	13 (13.7)	<0.001*
Frequency	45 (47.4)	6 (6.3)	10 (10.5)	<0.001*
Nocturia	47 (49.6)	7 (7.4)	2 (2.1)	
SUI	40 (42.1)	9 (9.5)	10 (10.5)	<0.001*
Dyspareunia	1 (1.1)	1 (1.1)	2 (2.1)	NA
**Anatomical objective outcome POP-Q points (Cm)**				
Ba	4.7±1.1	-2.9±0.4	-2.9±0.4	<0.001*
Bp	0.3±2.5	-2.5±1.2	-2.5±1.2	<0.001*
C	0.6±3.3	-6.3±0.9	-6.3±0.9	<0.001*

* All p values were statistically significant for the difference between status prior to surgery and 1 month following surgery.

No significant differences were found between 1 and 6 months following surgery.

4 patients had asymptomatic grade 2 rectocele (Bp = 1,1,0,0) at sixth follow-up visit.

**u**= Urinary Urgency; **f** = Urinary Frequency; **n** = Nocturia; **SUI** = Stress Urinary Incontinence; **BA** = B anterior POP-Q point; **C** = C POP-Q point; **BP** = B posterior POP-Q point

## DISCUSSION

Petros previously suggested ligamentary sling rather than large mesh implants for reinforcement for pelvic floor reconstruction (18). This is the first study looking at the possibility to minimize an in-use mesh implants surface area by skeletonization to a ligamentary harness rather than a large sheath mesh support, for potential reduction of mesh related complications, mainly erosion and pain, attributed significantly to the left over mesh mass.

The main findings of this study are that minimizing this mesh to a skeletonized ligamentary harness model for primarily reinforcement of the pelvic floor ligaments is feasible, effective and safe as the original mesh implant. These findings are attributed probably to the fact that a substantial fraction of the implant, affecting the pelvic soft tissue negatively and causing pelvic pain, might be not necessary for pelvic floor reconstruction reinforcement. The native pelvic floor supportive tissue architecture is basically ligamentary rather than a flattened fascial sheath, thus the mesh reinforcing implants should have the form of a “ligamentary pelvic harness” rather than a sheath. Most of the mesh implants adverse effects are likely related to excessive implanted mesh mass, thus shifting from large and high surface area implants (17, 19) to small surface area sling framework might very well reduce unwanted adverse effects.

Pain reduction is crucial when considering mesh implantation. It is especially important in the sexually active patient who might have dyspareunia after POP reconstruction.

We found no inferiority with the outcome among women who underwent vaginal reconstructive surgery with skeletonized partially absorbable mesh implants for the pelvic floor, regarding other intra-and post-operative adverse effects or pelvic floor dysfunction symptoms. The postoperative anatomical and subjective findings were similar as well.

This technique is not more complicated, neither more hazardous to perform than the common one.

This study strength is limited by being single armed and by having rather short-term follow-up. Further larger randomized controlled long-term studies should be carried out to shed more light on this important issue of minimizing the augmented mesh surface area and adapting the concept of ligamentary rather than a fascial sheath correction of pelvic organs prolapse. Although the particular mesh used in the present study is no longer available, the principal benefits and drawbacks of the skeletonized, ligamentary mesh implant harness are valuable and meaningful.

## CONCLUSIONS

Given that POP is a herniation process, one must acknowledge the importance of replacing the weakened fascia that caused the hernia defect with an implant to reinforce the reconstructive procedure, for assuring long-term cure. Yet, the surgeon must endeavor to reduce the mesh-related complications. This current study offers a new way for mesh implant adverse effects reduction, by adopting the skeletonized mesh concept.
